# Playing at the Schoolyard: “The Who’s, the What’s and the How Long’s” of Loose Parts

**DOI:** 10.3390/children10020240

**Published:** 2023-01-29

**Authors:** Joana V. Pereira, Jadiane Dionísio, Frederico Lopes, Rita Cordovil

**Affiliations:** 1 Centro Interdisciplinar de Estudo da Performance Humana, CIPER, Faculdade de Motricidade Humana, Universidade de Lisboa, Cruz-Quebrada, 1499-002 Lisbon, Portugal; 2 Laboratório de Comportamento Motor, Faculdade de Motricidade Humana, Universidade de Lisboa, Cruz-Quebrada, 1499-002 Lisbon, Portugal; 3 Faculdade de Educação Física e Fisioterapia, Universidade Federal de Uberlândia, Uberlândia 38400-678, Brazil

**Keywords:** free play, loose materials, physical properties, primary school children, affordances

## Abstract

Play has a key role in children psychomotor development, and the quality of play spaces can be a facilitator of the former. The physical properties of the environment, such as equipment or material available, can influence children’s behavior. However, it is not clear how the provision of different loose parts impacts children’s play patterns. This study aimed to analyze the influence of four types of loose parts on the time, frequency and number of children using them during free play sessions. We recorded the 1st, 5th and 10th sessions delivered by playworkers in a primary school, with 14 children (M_age_ = 9.96 years). The available loose parts were categorized, and four types of materials were chosen: tarpaulin/fabrics, cardboard boxes, plastic crates and plastic tubes. The influence of these materials on the time spent using them, frequency of use and number and sex of users (dependent variables) was analyzed. Some tendencies emerged, such as the popularity of tarpaulin/fabrics, but results showed no significant differences between materials. This could mean that the specific physical qualities of each loose part were not determiners of the behavioral domains analyzed. These findings suggest that all types of materials studied can be meaningful for children to engage with in diverse play opportunities.

## 1. Introduction

Play has a key role in children’s development, improving physical and mental health and wellbeing [1,2]. For instance, while playing freely, children have more opportunities to deal with novel and unexpected situations, learning how to cope with them [3]. They also solve problems and learn how to make decisions [4], and improve emotional competence, social functioning and cooperation [5]. Of equal importance, play enables children to produce and maintain a state of wellbeing as they test flexible behavior and generate other possible outcomes while simultaneously acquiring specific skills and cultural values [6,7,8]. For these reasons, psychomotricity practicians who work with children use play as a tool either for evaluation purposes or within the context of intervention [9]. There is even a method, based on children’s spontaneous action [10], that echoes the free play conceptualization. Thus, play also has a specific role in psychomotor development from a prophylactic point of view, considering that the psychomotricity perspective integrates physical, emotional, symbolical and cognitive aspects [11].

Although there is not a consensual definition of play [3,12], in the present research, we will address play as an activity particularly common during childhood that has no goal, is freely chosen, personally directed and intrinsically motivated [13]. In recent decades, we have witnessed a great decline in play opportunities during childhood, especially outdoors; this is related to changes in social lifestyle in today’s families, and to adults’ concerns about children’s academic achievements, particularly in western societies [4,14]. In this sense, it is critical to think about the quality of play spaces, which frequently do not meet children’s developmental needs and interests [15]. Parents, educators and policy makers have a key role in providing children with opportunities to play and assuring appropriate space and time for that purpose, as stated in Article 31 of the Convention on the Rights of the Child, reinforced by the General Comment no. 17 [16,17].

In order to better understand the transactional exchanges between children and their play environment from an ecological point of view [18], one of the aspects that should be investigated is how children interact with the physical characteristics of the outdoor spaces in which they play [19].

Previous studies, mainly with preschool children, indicate that the properties of the physical environment that are inherent to the landscape, the fixed equipment and material available to them have a direct connection to children’s preferences, and consequently to their behaviors [20,21]. For instance, children prefer vegetated yards and spacious areas with different landscape features to non-naturalized spaces [22], and the presence of natural vegetation on school playgrounds can be a potential predictor of children’s perceived restorativeness [23]. Some features of the physical environment can also work as facilitators for active play [24], and different playground facilities can have distinct associations with children’s physical activity at childcare [25]. Preschool children’s play behavior and consequently their different physical activity levels are associated with these fixed structures, but also with the portable play equipment available [26]. The links between environmental features and primary school children’s play behavior still need to be further explored.

Considering different types of environmental features, playgrounds are usually characterized by a type of surface (frequently rubber) and specific pieces of fixed equipment that often neither promote different types of play behavior nor afford novelty [27]. Adding loose parts to the playground has been suggested as a way for children to change the playground’s standardized structure by increasing their engagement and expanding the possibilities of creative actions [28], manipulating the playground to meet their play needs [29]. Loose parts, also called unstructured moveable materials or play props, are described as open-ended objects or materials, because they are not typical toys and do not have a specific play purpose [30]. They can be synthetic, such as ropes, boxes, tires or other waste materials, but also natural, like branches, sticks or leaves; they can be manipulated, moved, changed, and combined in different ways within children’s play [31].

Simon Nicholson proposed the Theory of Loose Parts, which states that the degree of creativity of an environment is directly proportional to the quantity and quality of existent variables [32]. He argued that by increasing and diversifying open-ended play resources such as loose parts, we can contribute to children adopting more flexible and novel actions, which in turn develop children’s imagination and creativity. In this sense, when children become more creative and imaginative in their play settings, their behavior and the setting in which the former occurs gain complexity, diversity and novelty.

Interventions with children in the school recess context regarding the provision of loose parts have already shown significant impact on different health domains, such as physical activity [33] and social development, and some emotional outcomes, such as enjoyment or self-esteem [34]. However, under a transactional person–environment approach, loose parts, due to their physical diversity in terms of form, size and type of material, have quite different physical properties, which may constrain and specify the way children interact with them [35]. Across this field, such specific interactions between the environment and the individual have been designated as affordances [36,37,38]. To the best of our knowledge, no previous study has yet addressed the issue of loose parts affordances in school children in terms of understanding the environmental specificities of such transactional relationships. A better understanding of the specific ecological context and nature of such interactions is needed to plan play interventions that can enrich children’s wellbeing and maximize developmental opportunities.

Thus, the present study, framed by ecological psychology and loose parts theories, followed a loose parts intervention, aiming to examine (1) how much time children spent using different types of loose parts, (2) how many children were engaged during that time with each type of loose part, and (3) to what extent those variables were associated with the physical qualities of the materials and with the progression of sessions, in an ecological free play context at the schoolyard. We hypothesized that the time spent using each loose part, the number of users and their sex, and the frequency of use were influenced by the physical properties of the loose part. We also hypothesized that children’s interaction with loose parts (regarding time spent using it, frequency of use, and the number and sex of users) would change across different play sessions.

## 2. Materials and Methods

### 2.1. Design

The present work is an observational longitudinal and exploratory piece of research on children’s play behavior. An ecological approach guided the current study, which stemmed from a set of loose parts play sessions provided by a play work collective in a schoolyard, between April and June 2021. Data were collected by the researcher in the 1st, 5th and 10th sessions.

### 2.2. Participants

Our sample comprised 3rd and 4th grade children from the participating school. The children (*n* = 14; 7 boys and 7 girls) were between 9 and 10 years old (M = 9.96; SD = 0.53), and all attended a mixed 3rd and 4th grade class. The participants’ backgrounds included a range of different nationalities and ethnicities, such as Portuguese, Brazilian, Indian, Pakistan, Ukrainian, and medium/medium-low socio-economic status of their families. The school was chosen as a convenience sample, as a consequence of the existence of a playwork intervention in the schoolyard, during a period with a lot of restrictions imposed by the pandemic situation. All children in the 3rd and 4th grade participated in the study.

### 2.3. Ethical Considerations

The Ethics Committee of Faculty of Human Kinetics, University of Lisbon, approved the project with the identification code 35/2020. Then, the main researcher contacted the school coordinator as well as the non-profit organization “1, 2, 3, Macaquinho do Xinês”, which was already organized and scheduled with the school coordination to deliver loose parts play sessions in the school play yard through playwork intervention. After having discussed the goals of each part, a schedule for the play sessions was set. Parents and caregivers provided informed consent after a meeting with the researcher, and children provided oral assent prior to data collection. Children were informed about the purpose of the research, how data would be collected, and that they could stop participating whenever they wanted.

### 2.4. Measures

Observational data were collected by video registration, using a fixed camera in the schoolyard (model GoPro HERO3) positioned two meters above the ground in a tree trunk. Additionally, a participant naturalistic observation was made by a researcher, who was in loco observing the children playing, and taking field notes regarding the play types and play narratives of children. Such data input will be considered in a future study. The social-demographic data were provided by the school coordinator, with caregiver’s consent.

### 2.5. Procedures

Before the beginning of the playwork intervention, the researcher responsible for data collection started attending the school recess to observe children while they were playing outdoors. This procedure allowed children to get used to her presence and to freely ask any question about the investigation. After six visits to the school, children were already apparently ignoring the presence of the observer. All data were collected having in mind the aim of not changing or interfering with children’s actions in the outdoor recess environment.

The playwork sessions were delivered by two playworkers from Associação 1, 2, 3, Macaquinho do Xinês. This is a non-profit organization that defends and promotes children’s right to play. The play sessions organized by this team rely on two main components: the tangible substrate of loose parts, described below, and also the intangible playwork principles [13]. This practice assures that play is free, child-led and not limited or oriented for a specific purpose (for further information, see Hughes, 2001 and Conway, 2007) [39,40].

The sessions had approximately one hour’s duration during the morning, and took place during spring and beginning of summer on a weekly basis. A total of ten play sessions occurred. However, this paper will only focus on three of them: the 1st, 5th and 10th. Besides the two playworkers and the researcher, the class teacher was also present in the schoolyard. The camera apparatus was always installed and turned on before children arrived so that interference was as minimal as possible. The materials were also placed in the schoolyard before children arrived.

### 2.6. The Outdoor Context

The public school where data were collected is located in Lisbon, but has some characteristics that are not very common among other urban schools. First, it has very few classes: one from kindergarten, one from 1st/2nd grade, and another from 3rd/4th grade. The aggregation of grades in the same class is due to the small number of students. Secondly, the school outdoor environment is very rich in vegetation and natural elements such as wild grass, bushes, big old trees, dirt, mud, etc. There are also many animals walking by, such as cats, peacocks, ducks and other birds. The view to the outside of the schoolyard fence is a public park, where people from the community walk their dogs and engage in some physical activity. The schoolyard has some wood structures, such as a train and two houses, but also two benches.

The Portuguese weather during this spring period varied between some rain sprinkles and blue sky, but the days on which the play sessions occurred had mainly clean weather. The temperatures on those days varied between 12–14 °C as a minimum and 19–25 °C as a maximum.

### 2.7. Materials

The loose parts that were made available for the play sessions were ropes and strings; bike tires and rims; car tires; cardboard boxes of different sizes; plastic crates; bins and bags; rags; tarpaulin and fabrics; plastic tubes of different lengths and shapes; cardboard tubes; soft sponge sticks; wood blocks; chalk; old keyboards; helmets; and pots, tupperware and other kitchen tools. Due to the variety of this equipment, for our research purpose, we grouped materials in categories based on their physical qualities and/or functions observed.

**Figure 1:**
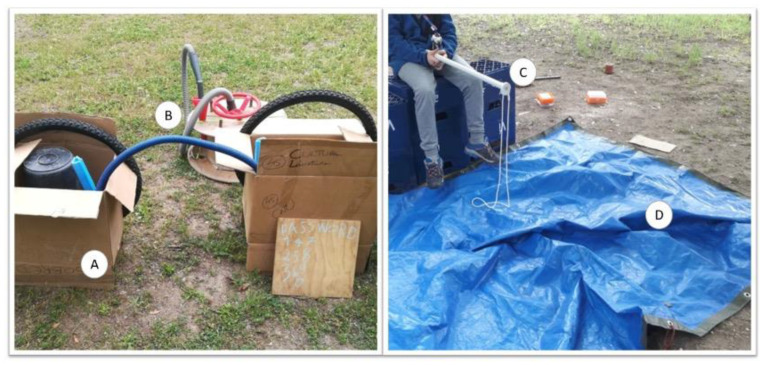
A. Cardboard boxes: flexible, changeable, lightweight, easily tearable; B. Plastic tubes: thin, long, flexible, unchangeable structure but changeable shape, lightweight; C. Plastic crates: rigid, heavy, unchangeable structure, stackable; D. Tarpaulin and fabrics: flexible, unchangeable structure but changeable shape, lightweight, and can occupy a large area.

For the present study, we choose to focus on four categories: tarpaulin or fabrics, cardboard boxes, plastic crates and plastic tubes. The criteria we used to make this choice were (1) the size of the material, which needed to be big enough to track in the video analysis, even if children were playing far away from the camera; (2) the number of elements available that belong to the same category, meaning that there were many units, and not only one specimen of that loose part; (3) the type of material should be present in each one of the three sessions; and (4) the types should differ considerably from each other regarding their physical qualities c.f., [32,36], as described in Figure 1.

### 2.8. Variables

The following variables were considered for analysis:

- Material—four types of loose parts varying in their physical qualities (Figure 1);
- Time with material—the time children spent using each loose part type, per session, in percentage;
- Frequency of use—number of times each material was touched, per session;
- Percentage of users—relative number of children that played with each material, per session.;
- Sex of users—percentage of boys and girls that played with each material, per session; and
- Session progression—over three time periods along the ten sessions, the1st, 5th and 10th.

### 2.9. Data Analysis

Video recordings (mp4 format, Codec H.264) captured at the schoolyard were uploaded in the ELAN software, Version 6.4, for the analysis [41]. With this software, the raters can visualize the recording and, at the same time, mark the beginning and end frame of each target behavior. This segmentation can be carried out using many tiers, representing the different layers of the observation which correspond in our case to the material types (four tiers). Thus, using ELAN, the visual data were transformed into time duration (msec) and frequency values that were extracted into XML format.

Interrater reliability was conducted taking in account 20% of total video duration. The inter-class correlation (ICC) values for the different variables were: ICC = 0.921 *p* = 0.033 for “play time”, ICC = 0.978 *p* = 0.005 for “frequency of use”, and ICC = 0.956 *p* = 0.014 for “percentage of users” and ICC = 0.962 *p* < 0.001 for “sex of users”. For the subsequent statistical analysis, SPSS 27 was used [42]. Descriptive statistics and frequency analyses were performed. Kruskal–Wallis tests were used to investigate the differences in play time, frequency of use and number of users between the different materials, with significance set at *p* < 0.05.

## 3. Results

### 3.1. Time Spent Using Each Material

**Figure 2:**
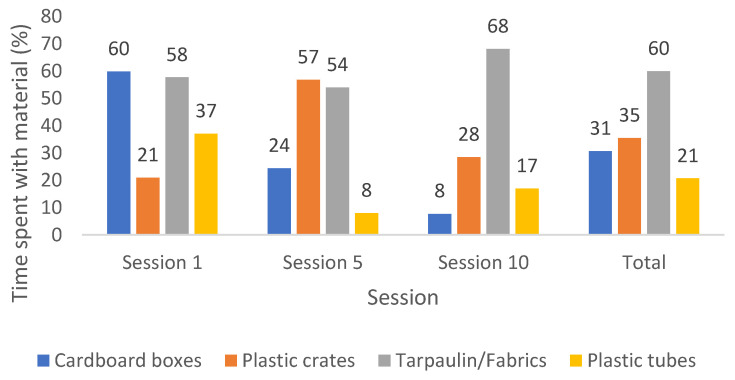
Percentage of time children spent playing with each material by session and in the total of the three sessions.

Considering the total time (see Figure 2), tarpaulin/fabrics was the type of material most used along the sessions (60%), followed by the plastic crates (35%), cardboard boxes (31%) and plastic tubes (21%). Although these percentages indicate that some materials were used more frequently than others, the differences were not statistically significant (H = 4.436, *p* = 0.218).

The material used for the most time varied in each session; in Session 1, the cardboard boxes were used 60% of the time, in Session 5, the plastic crates were used 57% of the time, and in Session 10, the tarpaulin/fabrics were used 68% of the time.

The usage time of the cardboard boxes showed a downward trend, decreasing progressively over the three sessions (60%, 24%, 8%). Cardboard boxes went from being the material used for the most time in the first session to the one used for the least time in the tenth.

As for the plastic tubes and plastic crates, the trend was neither clear nor stable. The type tarpaulin/fabrics had an increase in the time of use which was not continuous. That is, despite a small decrease of 4% in their time of use from the first to the fifth session, there was an increase from the first to the tenth session (from 58% to 68%).

### 3.2. Frequency of Use of Each Material

**Figure 3:**
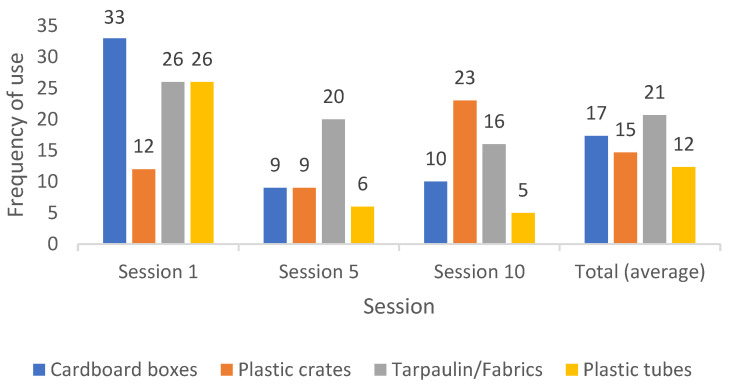
Frequency use of each material by session and average frequency in the total of the three sessions.

The type of material children used with more frequency during the play sessions (see Figure 3) was tarpaulin/fabrics, with an average of 21 times per session, and the plastic tubes were used less frequently, with an average of 12 times per session. However, the differences in the frequency of use of the different materials were non-significant (H = 1.191, *p* = 0.591).

The material used more frequently changed along sessions; in Session 1, the cardboard boxes were used more frequently (33 times), in Session 5, the tarpaulin/fabrics were used most frequently (20 times), and in Session 10, the plastic crates had the greatest frequency of use (23 times). The tarpaulin/fabrics had a progressive and constant decreasing trend in their frequency of use along the three sessions. The plastic tubes had a similar trend, but with a greater decrease between the first and fifth sessions. The frequency of use of the cardboard boxes also decreased, mainly from the first to the fifth session. The plastic crates were the only material that was used more frequently in the tenth than in the first session.

### 3.3. Percentage and Sex of Users

**Figure 4:**
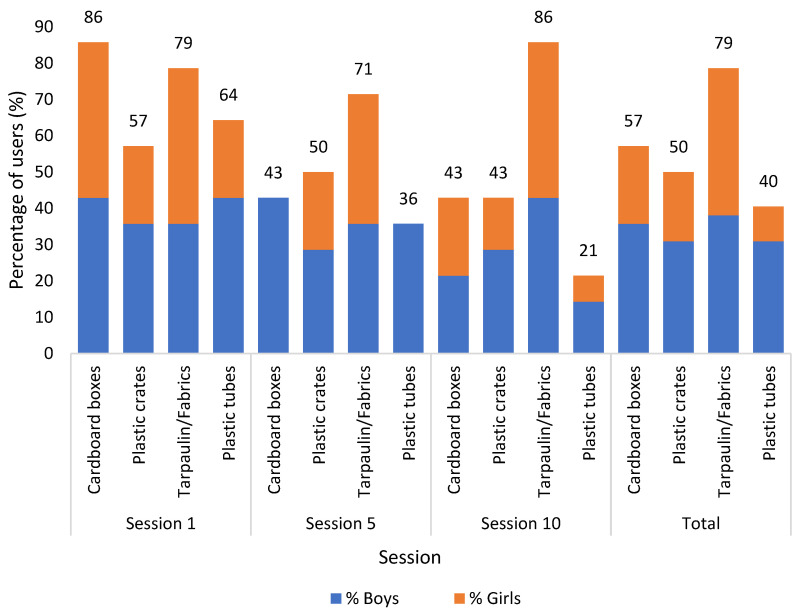
Percentage and sex of users per material, by session and in the total of the three sessions.

In the first session, all materials were used by more than 50% of children (see Figure 4). In the total of the three sessions, the tarpaulin/fabrics were used by most children (79%), followed by the cardboard boxes (57%), the plastic crates (50%), and finally, the plastic tubes (40%). The differences in the percentage of children using the different materials were not statistically significant for all children (H = 5.206, *p* = 0.157), neither for boys (H = 1.840, *p* = 0.606) nor girls (H = 5.710, *p* = 0.127).

The cardboard boxes were the material used by the most children in Session 1 (86%), but the tarpaulin/fabrics were more popular in Sessions 5 (71%) and 10 (86%). The most popular material in each session was always used by an equal number of boys and girls.

The number of children using the plastic crates decreased slightly over the sessions (from 57% to 43%). Plastic tubes were also used by fewer children along the sessions, but with a stronger decline (from 64% to 21%). The tendency was similar for the cardboard boxes, which were used by many children in the first session (86%), but this value decreased to half (43%) in the fifth session. Both the plastic tubes and the cardboard boxes were only used by boys in the 5th session. The tarpaulin/fabrics showed an opposite trend, as more children used them in the last session than in the first session (79% to 86%). In the last session, the tarpaulin/fabrics had twice as many users as the plastic crates and cardboard boxes.

## 4. Discussion

This study aimed to analyze the influence of four types of loose parts on the time, frequency and number of children using them during three sessions of free play. 

Regarding time of use, we hypothesized that the time spent using each loose part would be influenced by its physical properties. The descriptive results indicate that the tarpaulin/fabrics were used, on average, for a longer period of time than the other materials (i.e., almost three times more than the plastic tubes, and two times more than the cardboard boxes). This might indicate that the tarpaulin/fabrics have characteristics, such as size, lightness, malleability, and transformability, which allow their use in a broader range of play behaviors. However, the difference between the time of use of the four materials was not statistically significant, refuting our hypothesis. From an ecological approach to play, children perceive and act according to the existent affordances or possibilities for action [19,37], which depend on each child’s individual characteristics and on the characteristics of the physical environment [36], i.e., in this case, the different materials that child interacts with. Thus, loose parts with different physical qualities, textures, shapes, sizes and other particularities could have produced different affordances perceived by the children and, consequently, different behavioral patterns. Additionally, children’s intrinsic characteristics, such as their body features, experience, curiosity and creativity, might have also led them to explore the different loose parts in a varied way. According to our results, the different opportunities for action seemed to have been explored by the different children without a marked difference in the time of use of each loose part. Future studies, using larger samples, should try to confirm if the tendencies found in the descriptive analysis (e.g., more time using tarpaulin/fabrics) still remain non-significant. 

Regarding the differences in the time children used each material between sessions, we highlight two trends: the decreasing time of use of the cardboard boxes from the first to the last session, which contrasts with the increasing time of use of the tarpaulin/fabrics. This may mean that children perceived tarpaulin/fabrics as increasingly interesting materials, not running out of imagined uses for them in the first interactions (which highlights their open-ended type). On the other hand, children seemed to have lost interest in the use of the cardboard boxes, which might not be directly related to their greater interest in the tarpaulin/fabrics. The fact that the material used for the most time varied in each session (cardboard boxes, plastic crates, tarpaulin/fabrics) might indicate that all these materials have characteristics that are interesting or useful for children’s play, and this variability of affordances is important to promote children’s diverse play and creativity [32].

Concerning the frequency of use in relation to the way children use a certain material (either continuously or intermittently), the results indicate that there are no significant differences among the four types of loose parts, which does not support our hypothesis that children would interact more frequently with some materials than with others. The natural curiosity that characterizes primary school children might lead them to interact frequently with the different types of loose parts in the urge to explore them [43]. Despite the lack of significant differences, the descriptive data indicate that the number of interactions with the tarpaulin/fabrics was greater than the interactions with the other three materials. Thus, children picked up and dropped the tarpaulin/fabrics more often, reinforcing that there was a greater exploration of this material compared to the others, perhaps because it was perceived as more versatile due to its properties. 

Almost all materials, except for the plastic crates, had a decreasing trend in their frequency of use throughout the sessions. This tendency seems to reflect the exploratory behavior in the first session, in which children switched between materials more frequently; in the last sessions, they already knew the materials and probably chose the ones they wanted to play with without switching so frequently.

The transformable nature of loose parts [30,31] may explain the constant variability of the children’s behavior, due to the renewal of opportunities to change the environment, in this case through materials that are changeable and that can be combined almost infinitely. This effect of novelty is especially attractive because changes in the environment are made by children themselves, and not only presented to them [44]. Considering the individual factors, it is possible that the weekly frequency of the sessions was not sufficient for children of this age to create a specific preference for a certain material, maintaining the exploratory behavior typical of this developmental phase [45]. Future studies could analyze whether, with playwork sessions of a longer duration, more accentuated preferences would be formed. The same rationale is valid for increasing the session time beyond one hour, so that children could have the opportunity to engage in longer-lasting play cycles.

Concerning the number and sex of users, we found no significant differences among the number of children that used the different types of loose parts, and no material was significantly preferred over the others by boys or girls, thus not confirming our hypothesis. If children played equally with all materials, this might indicate that loose parts are sufficiently versatile and inclusive for all the play behavior that occurred, highlighting the potential of these materials to provide a variety of opportunities for action. Considering this, loose parts may play an important role in preventing the segregation of children into groups depending on what they play with.

Despite the lack of statistically significant differences, across all of the sessions, the tarpaulin/fabrics were the most popular material (i.e., used by the most children). This is in accordance with the results related to the time and frequency of use, highlighting once again the versatile nature of this material. The descriptive analysis also indicates that the number of users of the different loose parts tends to decrease throughout the sessions but increases for the tarpaulin/fabrics. The different trends between the number of children using the loose parts over the sessions may reveal the longitudinal evolution of the children’s preferences depending on the types of materials, which would be interesting to explore in more detail in future studies.

In the present study, the focus of research was exploring pertinent environmental transactional aspects of four types of loose parts affordances related to their physical specificities—the “time spent using specific materials”, “frequency of using specific materials” and “percentage of users of specific materials”—in a primary school yard which was under a playwork intervention. Our findings show that the specific physical qualities of each loose part type may not be significant determinants of the ecological behavioral domains that were analyzed. Nevertheless, descriptive analysis shows otherwise. Such ambivalence needs to be addressed, considering the ambiguous nature of play in itself [7], its unpredictable nature and expressional forms [16], and from the perspective of play as a deliberate creation of uncertainty which co-emerges as an entanglement of bodies, affects, objects, spaces and histories in ways that make life more pleasurable for the time spent playing [46].

### 4.1. Strengths, Limitations and Future Research

To our knowledge, this is the first study that has attempted to quantify environmental dimensions of transactional behavior using loose parts during children’s free play. As far as we are concerned, our study constitutes a novel methodological approach to the study of loose parts play. So far, most studies have focused more on the benefits of “loose parts play” for children in general, without trying to discriminate between the types of material or without characterizing behavioral changes in play patterns longitudinally.

Another strength of this study is that we have observed children in their natural environment, from an ecological perspective, while trying to quantify their behavior. This methodological approach had a large exploratory substrate, as we had few methodological references, and the studies that have addressed the relationship between these behavioral variables in play and the physical characteristics of the environment have focused more on structures than on loose parts [47,48].

The fact that our data are longitudinal allows a more comprehensive description of these phenomena. However, we are aware that our sample is very small and heterogeneous, and has a limited age range, which does not allow us to generalize the results, nor to use more robust statistical procedures. There are also other possible confounders that may have influenced the results, such as the fact that the camera could not capture all the schoolyard. Social variables were also not explored, and they also influence children’s behaviors. For instance, if a child who is seen as a leader uses a certain material, more children would probably be tempted to imitate him or her and use the same material.

It might be reductive to try to understand the child–environment transactions, more specifically, with the materials, based only on the analysis of the time and frequency of use and the number of users. In fact, we should not forget that these indicators do not give us clues about the quality and diversity of the actions developed. Hence, future studies should try to understand the complex nature of children’s use of the different materials.

### 4.2. Practical Implications 

Insights into children’s preferences and material choices allow us to better understand how we can respond to their needs, both from a developmental and wellbeing point of view, at the present time. Therefore, parents, education professionals, psychomotricians, and playworkers as well as designers and space planners should be aware of how environment influences children’s behavior, diversifying the resources and materials available for children to play with in order to give them a range of varied action opportunities.

## 5. Conclusions

The present study offered an innovative approach to the understanding of loose parts play and draws attention to the value of exploring specific environmental dimensions and aspects of playing as an ecological behavior that is an essential and default condition of children and childhood. Addressing the fundamental vectors of the emergence of affordances such as those integrated in the present study (“time spent using specific materials”, “frequency of using specific materials” and “percentage of users of specific materials”) opens up the possibility of generating valuable quantitative insights and critical reflections on children’s play, development and well-being.

Within the various loose parts analyzed in this study, none were absolutely preferred over the others, which leads us to conclude that none of those materials were “more ideal” than the others. In fact, our results show that all four types of the studied loose parts are meaningful for children to engage with in diverse play affordances, co-creating their play spaces in an environmentally meaningful way, exploring novelty and withholding their play narratives and frames. It is necessary to study in more detail the playful behavior of children in relation to the physical properties of their environment, namely through loose parts, to discover how they impact children and how we may respond to their needs and contribute to their health and wellbeing. 

In a child-led play environment, as in the one in which the present study occurred, when children have various loose parts to play with, the play setting is enriched and diversified. Loose parts enable children to shape and expand the environment according to their needs in relation to the available open field of affordances that may be included in their play cycles. This agency of action and behavioral flexibility, which reflects internal self-protection, resilience and emotional welfare, is of upmost importance for children’s wellbeing and development. This is crucial for all children’s lives and childhood in general, and it has a more supportive role in children that are accompanied by psychomotricians.

## Data Availability

The data presented in this study are available on request from the corresponding author. The data are not publicly available due to privacy or ethical restrictions.
